# Incentivizing access to family planning in Senegal via the informed push model

**DOI:** 10.1186/2052-3211-7-S1-O12

**Published:** 2014-12-17

**Authors:** Leah Hasselback, Babacar Gueye, Oumy Ndao, Soussaba Kanoute Ndour, Carol Cissé

**Affiliations:** 1 IntraHealth International, Washington DC, USA

## Background

In Senegal, the absence of a well-functioning family planning (FP) product supply chain has acted as a significant supply-side barrier and contributed to Senegal’s low contraceptive prevalence rate (CPR) (12.3% in 2010) and high unmet need for FP among married women (29%). Recurrent FP product stockouts at nearly 80% of public service delivery points (SDPs) continue to hinder the government’s ability to achieve its goal of doubling CPR to 27% by 2015.

## Method

In 2012, IntraHealth International conducted a pilot of the informed push model (IPM) in 2 Senegalese regions to improve family planning product distribution. IPM, a last-mile distribution mechanism, moves FP products monthly from national pharmacy depots to health facilities via dedicated private logistics professionals. By utilizing task shifting, IPM reduces stockouts, which allows health workers to focus on health service provision and client satisfaction. The initiative is currently being expanded nationally.

## Results

IPM immediately reduced and maintained stockout levels below 2% throughout the six-month pilot period. In target districts, contraceptive consumption increased by 38% and key logistics data reporting rose dramatically from 0% to 100%. As IPM is being scaled up, health workers in the eight regions already utilizing the model have described it as a “revolution.” At the health facility level, clients are benefitting from a constant supply of FP products and increased focus on provider-client interactions, resulting in more satisfaction with FP services received. Providers have also expressed greater job satisfaction, improved work flow, and better-quality reporting of data.

## Discussion

Improvements in the FP commodity supply chain have the potential to boost health worker retention, improve client satisfaction, and increase women’s access to contraceptives. With IPM, the logistics management burden is shifted from health workers to dedicated logistics professionals, leaving more time for providers to focus on service delivery quality. The model reintroduces a cost recovery system, which makes funds available so providers can ensure that clients have access to the methods they want. IPM also strengthens the supervision system by providing health workers with the opportunity to clarify their roles and responsibilities and improve workflow at the facility level.

## Lessons learned

Shifting non-medical tasks from health providers to logistics professionals improves the service quality and provides women with a constant supply of family planning products. IPM strengthens public-private partnerships while incentivizing all parties to ensure that facilities and communities have access to family planning products.

